# The stabilizing potential of the supraspinatus is inhibited in tear-associated scapula shapes but can be modulated by kinematic adjustments

**DOI:** 10.3389/fbioe.2025.1505015

**Published:** 2025-03-31

**Authors:** Erin C. S. Lee, Nathan M. Young, Ellen Y. Li, Rebekah L. Lawrence, Michael J. Rainbow

**Affiliations:** ^1^ Department of Mechanical and Materials Engineering, Queen’s University, Kingston, ON, Canada; ^2^ Department of Orthopaedic Surgery, University of California, San Francisco, San Francisco, CA, United States; ^3^ Program in Physical Therapy, Washington University School of Medicine, St. Louis, MO, United States

**Keywords:** scapula shape, rotator cuff tears, glenohumeral stability, supraspinatus, musculoskeletal model, morphology

## Abstract

**Introduction:**

Specific scapula shapes are associated with full-thickness tears of the supraspinatus tendon. A primary role of the supraspinatus is to actively stabilize the glenohumeral joint against muscles that generate destabilizing shear forces. Mechanisms that increase the supraspinatus load required to perform this stabilizing function may increase an individual's susceptibility to tears. Previous work has shown that tear-associated scapula shapes increase the destabilizing action of the deltoid during arm-raising, but no work has investigated whether tear-associated shapes inhibit the stabilizing potential of the supraspinatus itself.

**Methods:**

We combined statistical shape modeling, kinematics-driven simulations of the glenohumeral joint, and a finite element model of the supraspinatus to investigate the interactions among shape, kinematics, and the stabilizing potential of the supraspinatus. First, we identified tear-associated 3D scapula shapes using partial least squares discriminant analysis. Second, we examined how tear-associated shapes alter the stabilizing potential of the supraspinatus given the same kinematic path. Finally, we examined the extent to which kinematic perturbations could modulate differences in stabilizing potential.

**Results:**

Relative to asymptomatic controls, individuals with full-thickness tears possessed a suite of 3D shape differences including narrower supraspinous fossae and anteverted glenoids. For the same abduction path, tear-associated scapula shapes caused supraspinatus fibres to act more anteriorly and less compressively compared to the control shapes, potentially inhibiting the supraspinatus' ability to stabilize the humeral head. When the abduction path of the tear-associated scapula was internally rotated and shifted anteriorly, the supraspinatus line-of-action closely resembled that of the control-associated scapula; however, the tear-associated shape still possessed a narrower breadth in its supraspinatus line-of-action.

**Discussion:**

Our findings indicate that tear-associated scapula geometry may inhibit the stabilizing potential of the supraspinatus, but this shape-driven change could be partially modulated when the abduction path of the tear-associated shape was perturbed. The magnitude of kinematic perturbations required to modulate function exceeded the magnitude of shape differences, indicating that the perturbations are not correcting for a simple offset, but rather complex changes in muscle geometry that occur due to 3D shape differences.

## 1 Introduction

Full-thickness tears (FTTs) of the rotator cuff affect 20%–23% of the population and most frequently involve the supraspinatus tendon (Minagawa et al., 2013; Tempelhof et al., 1999; Yamamoto et al., 2010). While the pathways to injury are complex, chronic tendon overload is likely an important contributor to degenerative tears (Lawrence et al., 2024; Seitz et al., 2011). As a rotator cuff muscle, a primary role of the supraspinatus is to actively stabilize the glenohumeral joint against muscles that generate destabilizing shear forces on the humeral head (Lee et al., 2000; Lippitt et al., 1993). Human scapula shape is highly diverse (Dwight, 1887; Graves, 1921) and specific scapula shapes are associated with supraspinatus FTTs (Banas et al., 1995; Bigliani et al., 1986; Hughes et al., 2003; Moor et al., 2013; Nyffeler et al., 2006; Tétreault et al., 2004). Biomechanical models have indicated mechanisms through which FTT-associated features increase the supraspinatus load required to perform its stabilizing function. For example, a larger critical shoulder angle and a more superiorly inclined glenoid increase the destabilizing action of the deltoid, thus increasing the load in the supraspinatus required to maintain glenohumeral stability during abduction (Gerber et al., 2014; Moor et al., 2016; Viehöfer et al., 2015). While previous work has focused on shape-mediated changes to the destabilizing force of the deltoid, no work has investigated how scapula shape alters the line-of-action and stabilizing potential of the supraspinatus itself. Three-dimensional (3D) shape features associated with FTTs include variation in the shape of the supraspinous fossa, indicating potential changes to the muscle’s line-of-action (Lee et al., 2020). If FTT-associated scapula shapes inhibit the supraspinatus’s stabilizing potential, individuals with those shape features may experience higher tendon loads to perform the same shoulder function as those without FTT-associated features. This information may contribute to our understanding of the etiological mechanisms of supraspinatus tears.

Previous studies identifying FTT-associated shapes are limited in their ability to assess how variation in shape alters the supraspinatus’s stabilizing potential. While discrete metrics like the critical shoulder angle effectively distinguish individuals with FTTs from asymptomatic controls (Moor et al., 2014), they do not capture 3D changes to rotator cuff attachment sites. Rather, 3D statistical shape models enable morphable musculoskeletal models that account for changes across the whole bone (Clouthier et al., 2019). Previous 3D shape analyses have used unsupervised dimension reduction (i.e., Principal Component Analysis) to identify modes of scapula shape variation without *a priori* assumptions on group differences (Lee et al., 2020). While Principal Component Analysis can identify group differences in shape, these differences are often distributed across many shape modes. Supervised techniques, such as Partial Least Squares, make assumptions on group structure that can help formalize and identify shape differences between clinically relevant groups (e.g., tear status). Partial Least Squares can therefore reveal modes of variation more relevant to tendon pathology (Clouthier et al., 2023) and can be used to generate theoretical 3D scapula shapes that isolate FTT-associated features. The influence of FTT-associated shape changes on shoulder mechanics can then be investigated with a musculoskeletal model.

While musculoskeletal models have been used to examine shoulder mechanics, existing models are not well-suited for capturing 3D shape-related changes to the broad, fan-like orientation of the supraspinatus fibres. Previous models represent the supraspinatus as a single fibre (Lee et al., 2020) and/or use idealized wrapping surfaces that do not capture changes to bony geometry (Garner and Pandy, 2001; Saul et al., 2015). Finite-element models and multi-fibre models that do capture the breadth of the supraspinatus are not readily morphable across scapula shapes (Hoffmann et al., 2017; Péan et al., 2019; Péan and Goksel, 2020; Webb et al., 2014). Based on the limitations of these previous models, we believe that multi-fibre, morphable models are well-suited for assessing how the supraspinatus’ broad line-of-action and stabilizing potential are altered by 3D shape changes.

The effect of scapula shape on muscle function has been previously explored under the assumption that rotational kinematics are identical across shapes (Lee et al., 2020; Viehöfer et al., 2015). However, shoulder girdle kinematics are highly variable *in vivo* (Kolz et al., 2021), and many individuals have at-risk shapes and healthy tendons (Lee et al., 2020). Although this protection from pathology is undoubtedly multifactorial, individuals with at-risk shapes may modulate muscle function by adapting their kinematics according to their unique anatomy. Approaches that account for kinematic variability can explore whether shape-driven changes in muscle function can be mediated by kinematic adjustments.

In this study, we combine a scapula shape model, kinematics-driven simulations of the glenohumeral joint, and a finite element model of the supraspinatus to investigate the interactions among shape, kinematics, and the stabilizing potential of the supraspinatus. The objectives of our study were to (1) identify FTT-associated 3D scapula shapes using supervised dimension reduction, (2) examine how FTT-shapes alter the stabilizing potential of the supraspinatus given the same kinematics, and (3) examine the extent to which kinematic perturbations could modulate differences in stabilizing potential. We hypothesized that (1) FTT-associated 3D scapula shapes would encompass discrete shape features previously reported in the literature, (2) FTT-associated shapes would inhibit the stabilizing potential of the supraspinatus, and (3) the shape-driven differences in stabilizing potential could be modulated with kinematic adjustments.

## 2 Methods

### 2.1 Overview

**FIGURE 1:**
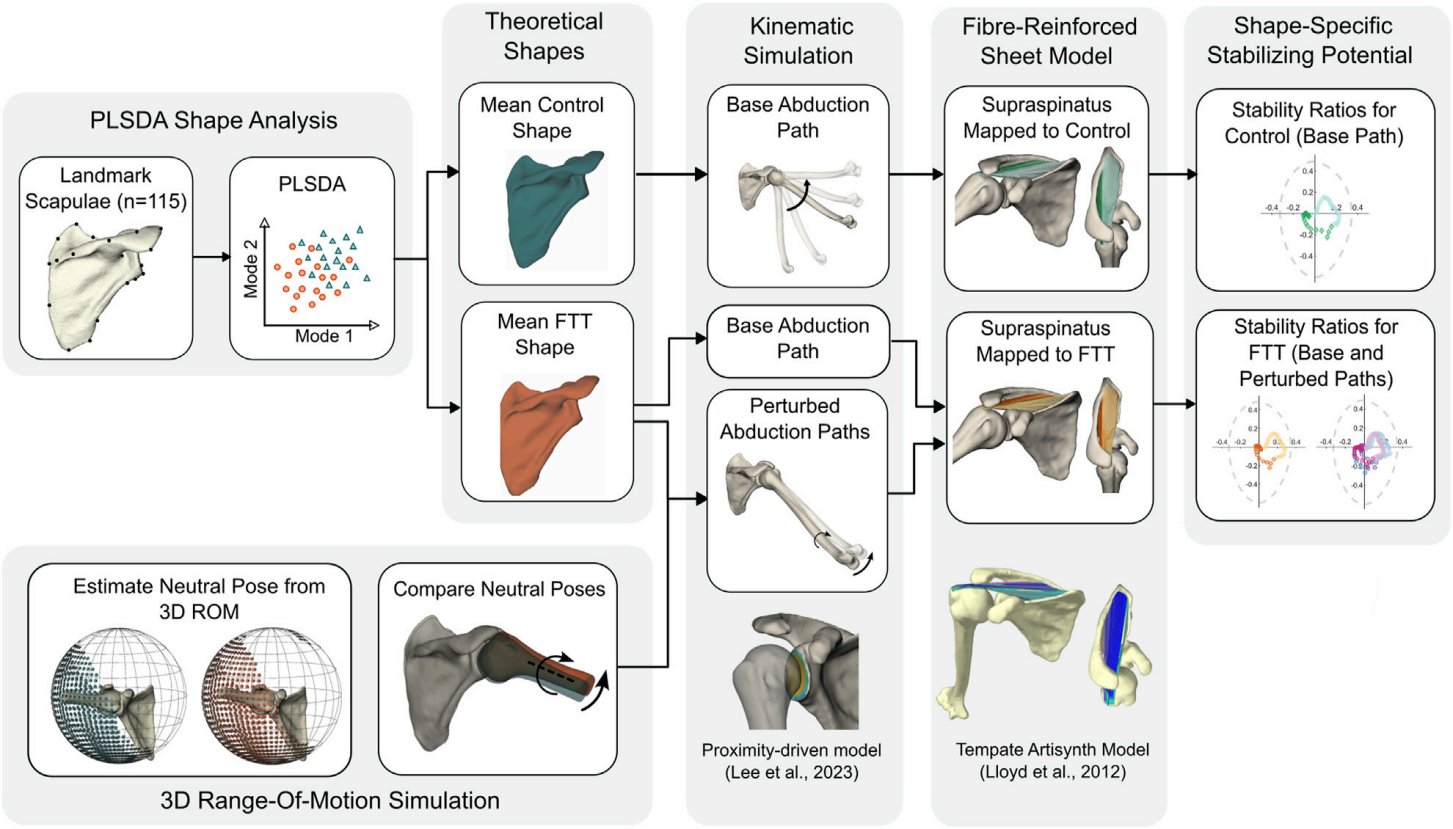
Flowchart of methodological approach.

Our approach combined statistical shape modeling, kinematic simulation, and a morphable musculoskeletal model to investigate shape-driven changes to the stabilizing potential of the supraspinatus (Figure 1). We first developed a Partial Least Squares Discriminant Analysis (PLSDA) model to generate control- and FTT-associated scapula shapes. Using a proximity-driven glenohumeral model (Lee et al., 2020), we then simulated a base (i.e., standardized) abduction kinematic path for both the control- and FTT-associated shapes. We also simulated perturbed abduction kinematics for the FTT-associated shape, which were informed by each shape’s relative kinematic neutral pose (Lee et al., 2023). Finally, we modelled the supraspinatus as a set of fibre-reinforced finite element sheets on a template model and mapped this onto the control- and FTT-associated shapes and their simulated kinematic paths. Supraspinatus stabilization potential was quantified by the stability ratio of each muscle fibre.

### 2.2 Identifying FTT-associated scapula shapes

We developed a landmark-based statistical shape model from 115 scapula meshes segmented from computed tomography scans (age = 57 ± 6 years, 40 males and 75 females). This sample combined three datasets collected at the University of Utah (Kolz et al., 2021; Kolz et al., 2020) and Henry Ford Health (Bey et al., 2009; Bishop et al., 2009; Lawrence et al., 2024; Lee et al., 2020). This sample is a subset of those described in Lee et al. (2024), retaining only individuals over the age of 45 years. The sample included 24 individuals with symptomatic FTTs of the supraspinatus (confirmed by MRI or ultrasound) and 91 asymptomatic controls. The asymptomatic group included individuals with no structural tears, individuals with partial-thickness tears, and individuals with full-thickness tears. Since we previously found that males and females possessed significantly different scapula shapes (Lee et al., 2024), we performed a two-proportions Z-test to ensure that the proportion of sex did not differ between groups (FTT: 71% female, control: 64% female, χ^2^ = 0.17, p = 0.68).

To capture the 3D shape of each scapula, we identified 29 homologous landmarks capturing the geometry of articular sites and muscle attachment areas (Lee et al., 2024; 2020). A single observer semi-automatically digitized all landmarks in Stratovan Checkpoint (*Stratovan Corporation*, Sacramento, United States). A previous intra-observer error analysis for this process confirmed that the magnitude of shape differences across individuals exceeded the magnitude from intra-observer error (Lee et al., 2024). We performed Procrustes superimposition to scale, rotate, and align all landmarks to a common size and orientation, yielding 3D Procrustes shape coordinates that describe only variation in shape. Procrustes superimposition was performed with the *R* package, *geomorph* (Adams et al., 2023; Baken et al., 2021).

To identify and assess shape modes that differentiate individuals with symptomatic FTTs from asymptomatic controls, we used PLSDA. While Principal Component Analysis is an unsupervised technique that yields shape modes that explain successively smaller amounts of variation in shape, Partial Least Squares (PLS) is a supervised technique that identifies modes that explain successively smaller amounts of *co-variation* between a predictor block, *X*, and a response block, *Y* (Zelditch et al., 2012). Here, the predictor block, *X*, consisted of the 3D Procrustes shape coordinates. The response block, *Y*, consisted of group labels where symptomatic FTTs were assigned a response score of *y* = 1 and controls were assigned a score of *y* = 0. PLSDA is a form of PLS regression that identifies an *m*-dimensional discriminant axis along *m* shape modes that best differentiates groups. In other words, PLSDA allows us to specifically explore variability in scapula shape that is associated with supraspinatus pathology status.

Since PLSDA is prone to overfitting (Ruiz-Perez et al., 2020), we performed cross-validation to carefully select the optimal number of retained modes, *m*, and assess model performance. We first generated an initial PLSDA model that retained *M* modes explaining 70% of the total variation in shape. We then recomputed *M* PLSDA models, varying the number of retained modes from 1 to *M*. We performed internal leave-one-out cross-validation where, in each model, we systematically excluded individuals from the model and computed a predicted response score (*y*) from their 3D shape coordinates (Clouthier et al., 2023). If the predicted response score was *y* > 0.5, they were classified as a symptomatic FTT. If the predicted response score was *y* < 0.5, they were classified as a control. We then assessed each model’s performance by comparing the predicted labels to the true labels. Since the group sizes were unbalanced, we summarized model performance with balanced accuracy, F1 score, and geometric mean (G-mean). Balanced accuracy is the arithmetic mean of sensitivity (true predicted FTTs among all FTTs) and specificity (true predicted controls among all controls). F1 score and G-mean are, respectively, the harmonic and geometric means of precision (true predicted FTTs among all predicted positives) and recall (true predicted FTTs among all FTTs; equal to sensitivity). We considered the optimal number of retained modes, *m*, to be those retained in the PSLDA model that yielded the highest balanced accuracy.

To determine if our PLSDA model performed better than a PLSDA model generated from groups with no underlying difference (null model), we followed the methodology of Clouthier et al. (2023). We generated 20 samples with randomly assigned labels, retaining the same group proportions as in the true sample. We computed a PLSDA model for each model following the same process described above (including identifying the optimal number of modes). We compared the performance of the true sample’s PLSDA model to that of the random samples’ PLSDA models, yielding a Z-score and p-value for balanced accuracy, F1 score, and G-mean. We considered the true model to perform better than the random models if p < 0.01 across all performance metrics. We used MATLAB 2023b (*Mathworks*, Natick, United States) to generate and assess all PLSDA models.

We then used thin plate spline warping to generate control- and FTT-associated scapula shapes for visualization and further biomechanical modelling. With thin plate spline, a template mesh is warped such that its landmark coordinates match the landmark coordinates of the target mesh and the surface between landmarks is interpolated to minimize bending energy (Bookstein, 1989). Therefore, the surface between landmarks of the resulting warped mesh retains similar characteristics and curvature of the template mesh. We first identified a template mesh by selecting the scapula of the individual with the lowest Procrustes distance from the mean mesh (i.e., the lowest square root of the sum squared distances between corresponding landmarks). We then generated a mean mesh by warping the template mesh to match the mean Procrustes coordinates. Finally, we warped the mean mesh to generate a control-associated shape (the shape associated with a response score of *y* = 0), and an FTT-associated shape (the shape associated with a score of *y* = 1). These shapes are also equivalent to the mean shapes of all scapulae in the control group and FTT group, respectively, projected onto the *m* retained modes of variation. The control- and FTT-associated Procrustes shape coordinates were computed in MATLAB, and the thin plate spline warps were generated in *R* with *geomorph* (Adams et al., 2023; Adams et al., 2013).

To quantitatively compare our FTT-associated scapula shape features to those reported in the literature, we calculated the following discrete metrics on the mean control shape and mean FTT shape using custom MATLAB scripts (Lawrence, 2023): critical shoulder angle (Moor et al., 2013), glenoid inclination, glenoid version, glenoid height, glenoid width, acromion coverage (Beeler et al., 2018), and lateral acromion ratio which measures the extent of lateral acromion overhang. The computation for each metric is described in the Supplementary Material (Supplementary Table S1).

### 2.3 Kinematic simulation

We generated kinematics-driven musculoskeletal simulations for each scapula shape using our previously developed proximity-driven glenohumeral model (Lee et al., 2023; Lee et al., 2020). To isolate the effect of shape, we first simulated identical, base kinematic paths that approximated the glenohumeral motion observed during scapular plane abduction in a full-can (“thumb-up”) position. Informed by average glenohumeral kinematics measured with biplanar videoradiography, we simulated 10°–100° of glenohumeral abduction in an elevation plane 5° anterior to the scapular plane with 50° of external rotation (Kolz et al., 2021; Ludewig et al., 2009). At 5° increments in abduction, we used the proximity-driven model to optimize joint translations at the given rotational pose (Lee et al., 2020). We paired each scapula shape with the same template humerus belonging to the individual with the scapula shape closest to the mean. We defined scapula and humerus coordinate systems such that the x-axis was oriented laterally, the y-axis was oriented superiorly, and the z-axis was oriented posteriorly, consistent with Lee et al. (2020). We used the International Society of Biomechanics (ISB)-recommended coordinate system for the humerus (Wu et al., 2005). For the scapula, we adapted the ISB recommendations such that the superior-inferior axis was aligned with the medial border, the anterior-posterior axis was normal to the scapular blade, and the medial-lateral axis was defined as their cross-product. We generated 3D rotational poses by defining rotations in a Y-Z-Y Euler sequence: elevation plane, abduction, and axial rotation.

We generated perturbed kinematics paths for the FTT-associated shape to examine the potential effect of kinematic adaptations. We perturbed the base path by 5° and 10° in elevation plane and axial rotation independently and in combination. To determine the direction of perturbation, we compared the FTT shape’s kinematic neutral pose to the control shape’s neutral pose. We estimated each shape’s kinematic neutral as the centroid of its 3D glenohumeral range-of-motion simulated with the proximity-based model (Lee et al., 2023).

### 2.4 Supraspinatus model

To capture geometric changes in the supraspinatus line-of-action throughout motion and across shapes, we modelled the supraspinatus for each shape in the open-source modelling package, Artisynth (Lloyd et al., 2012). On the sample’s mean mesh, we first digitized points defining the perimeter of the supraspinatus origin and insertion sites on the scapula and humerus, respectively. As the thin plate spline maintains point correspondence across warped meshes, we automatically mapped the origin points onto the control- and tear-associated scapulae, again using the template humerus for both models. This process removes any variability due to repeated manual point digitization.

**FIGURE 2:**
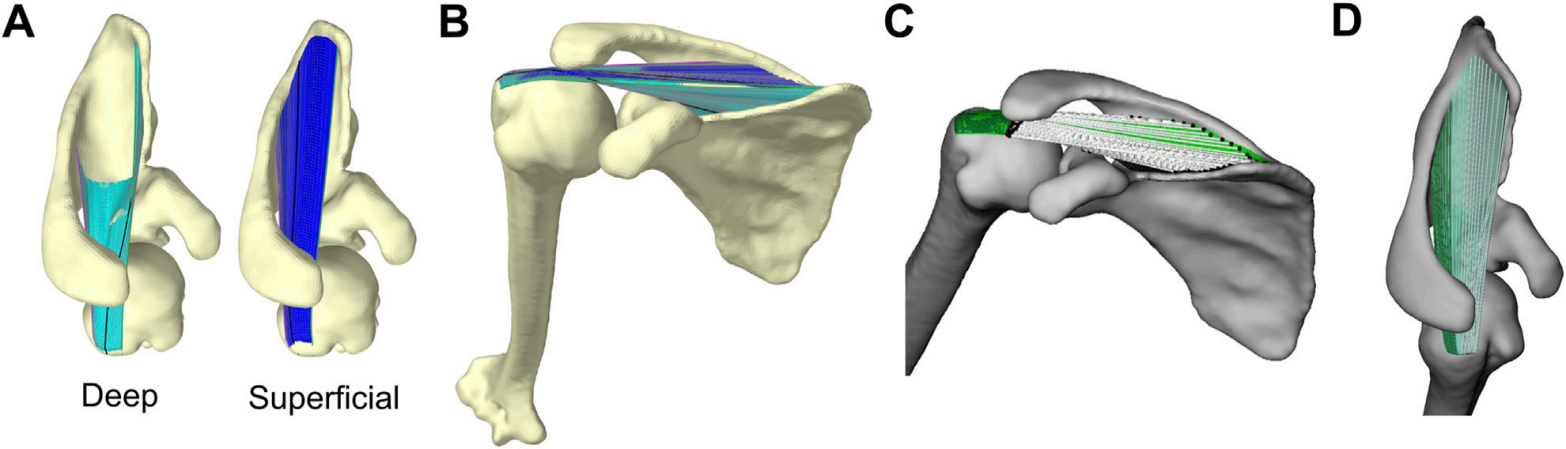
Artisynth model of supraspinatus and glenohumeral joint **(A)** Deep (left, cyan) and superficial (right, blue) sheets that together capture the entire perimeter of supraspinatus origin site on scapula **(B)** Full model where fibre-reinforced sheets wrap around bony surfaces **(C)** The fibres’ lines-of-action visualized by white lines connecting each fibre’s effective origin (black spheres on scapula) to effective insertion (black spheres on humerus). The line-of-action is the unit vector oriented along the white lines from origin to insertion. The finite-element sheets are plotted in green **(D)** Supraspinatus anterior (light) and posterior (dark) subregions.

For each shape, we modelled the muscle as two finite-element sheets using the LinearMaterial class in Artisynth. We used deep and superficial sheets to capture the entire perimeter of the broad supraspinatus origin, effectively forming a tube representing the muscle’s exterior (Figures 2A, B). We reinforced each sheet with longitudinal fibres running from origin to insertion using Artisynth’s LinearAxialMuscle class. The deep sheet was wider and reinforced with 30 fibres equally spaced along the perimeter, while the superficial sheet was reinforced with 20 fibres. All fibres were defined by 100 nodes from origin to insertion.

The fibres and finite-element sheets were constrained to wrap around the bony surface of the scapula and humerus. Since our simulations were kinematically-driven, the purpose of the fibre-reinforced sheets was to capture the breadth of the supraspinatus and prevent the fibres from wrapping independently from one another. Therefore, we did not need to implement physiological material properties that yield accurate estimates of stress or strain. Instead, in an iterative process, we manually determined material properties (e.g., stiffness, damping, Poisson’s ratio) that enabled realistic geometric wrapping: we ensured the sheets provided adequate stiffness to keep the fibres close together (i.e., did not spread apart while wrapping around the convex surfaces like the humeral head; see Supplementary Figure S1), that all elements in the sheet elongated or shortened at similar rates (rather than a few elements accounting for most of the muscle’s elongation), and that all fibres and sheets remained in tension throughout the entire range-of-motion. We used the same material properties for all scapula shapes and kinematic paths.

### 2.5 Quantifying stabilizing potential

We quantified stabilizing potential using stability ratios, an intuitive metric for describing how the tensile force in a muscle would act to translate the humerus relative to the glenoid (Ackland and Pandy, 2009; Mulla et al., 2020). We computed stability ratios for all fibres to capture the breadth of the supraspinatus’ action in a given rotational pose. We first identified each fibre’s effective origin and insertion point as the fibre nodes that first contact the scapula or humerus, respectively (Pandy, 1999). Each fibre’s line-of-action (its force-direction acting on the humerus) was the unit vector oriented from the effective insertion to the effective origin (Figure 2C). We then resolved each line-of-action into a glenoid-based coordinate system defined by the principal axes fit to the points along the perimeter of the glenoid surface (Lawrence et al., 2022). The glenoid’s medial-lateral axis was the principal axis directed compressively into the glenoid, the anterior-posterior axis was the principal axis directed anterior-posteriorly along the glenoid, and the superior-inferior axis was the principal axis directed superior-inferiorly along the glenoid. We then computed each fibre’s superior-inferior (SI) and anterior-posterior (AP) stability ratios, which describe the shear component of the line-of-action ( $f_{shear,SI}$ in SI or $f_{shear,AP}$ in AP) divided by the compressive component of the line-of-action ( $f_{compressive}$ ):

$$SI\;Stability\;Ratio=\frac{f_{shear,SI}}{f_{compressive}}$$

$$AP\;Stability\;Ratio=\frac{f_{shear,AP}}{f_{compressive}}$$

where $f_{shear,SI}$ is positive superiorly and negative inferiorly, and $f_{shear,AP}$ is positive anteriorly and negative posteriorly. For each stability ratio, a higher magnitude indicates a greater tendency to cause shear translation of the humeral head. Across both sheets, we grouped the fibres into anterior and posterior regions to represent their distinct anatomical compartments (Figure 2D) (Kim et al., 2007; Roh et al., 2000).

## 3 Results

### 3.1 PLSDA model and FTT-associated scapula shapes

**FIGURE 3:**
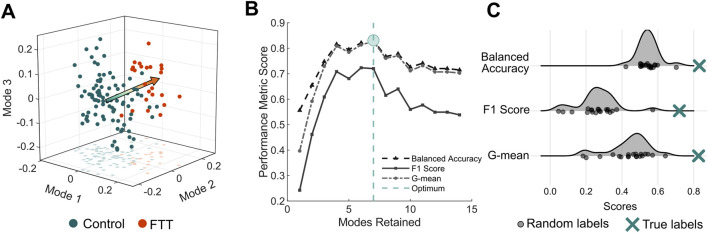
PLSDA model performance **(A)** Individual scapula shapes projected onto the first three modes of the PLSDA model. The arrow is oriented from the mean control shape (y = 0) to the mean FTT shape (y = 1) **(B)** Performance metrics as a function of modes retained in the PLSDA model. As fourteen modes were required to explain 70% of the total shape variation, we generate PLSDA models retaining one to fourteen modes. The model with the highest balanced accuracy indicated the optimal number of modes **(C)** Comparison of performance of PLSDA models generated from twenty samples with random labels to the PLSDA model generated from the true sample labels.

The PLSDA model retaining seven shape modes achieved the highest balanced accuracy for classifying scapulae belonging to individuals with symptomatic FTTs from asymptomatic controls (Figure 3). The first seven modes cumulatively explained 48% of the total shape variation. The final PLSDA model’s balanced accuracy was 83%, the F1 score was 72%, and the geometric mean was 83%. The PLSDA model generated from the true sample performed significantly better than the models generated from the randomly labelled samples for all performance metrics (Z > 3.3, p < 0.001).

**FIGURE 4:**
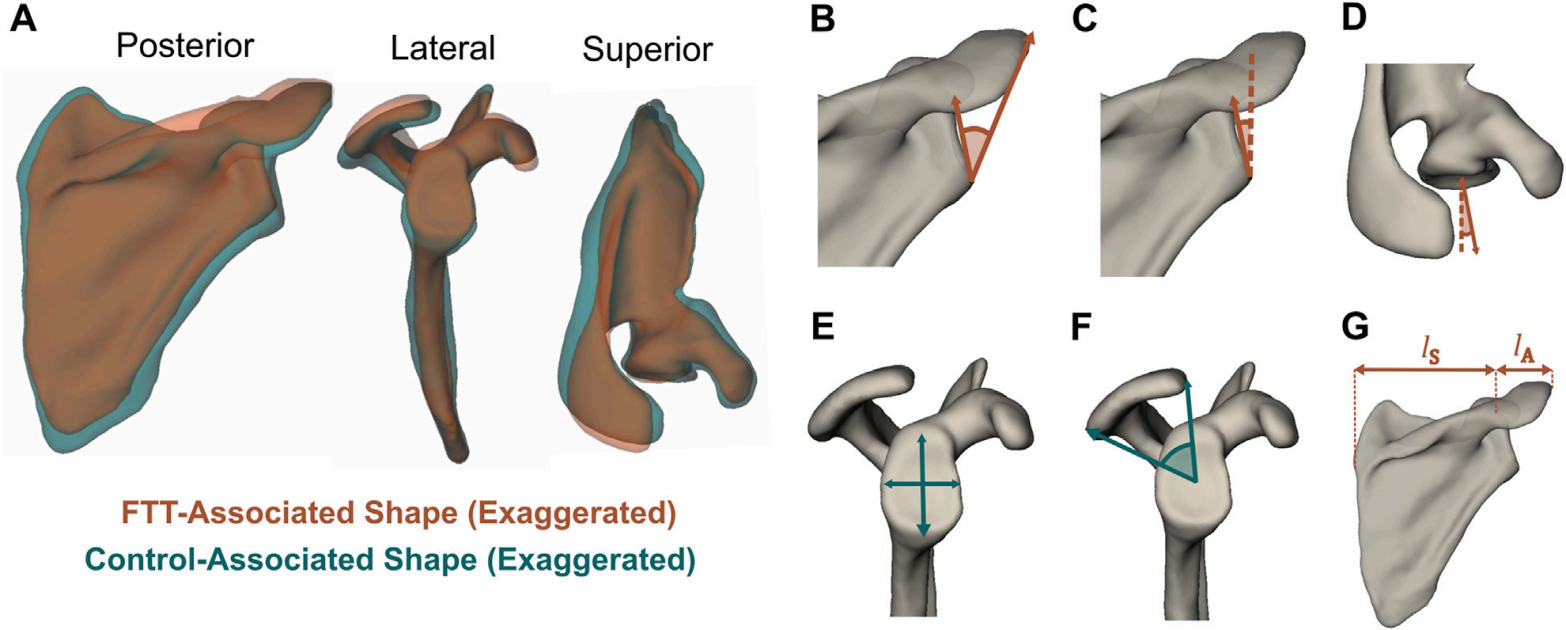
**(A)** FTT-associated and control-associated shapes generated from thin plate spline warps with shape differences exaggerated. The visualized control shape corresponds to y = −1 and the visualized FTT shape corresponds to y = 2, meaning the magnitude of shape difference here is three times larger than the magnitude between the mean FTT shape and mean control shape. The mean FTT shape and mean control shape are available as 3D meshes in the Supplementary Material **(B–G)** Discrete metrics visualized on the mean FTT shape. Metrics that are greater on the FTT shape are in orange, while metrics that are greater on the control shape are in teal **(B)** Critical shoulder angle (Moor et al., 2013) **(C)** Glenoid inclination **(D)** Glenoid version **(E)** Glenoid height and width **(F)** Acromion coverage (Beeler et al., 2018) **(G)** Lateral acromion ratio = l_A_/l_S_ × 100%.

FTT-associated shape exhibited significant differences in 3D morphological features relative to the control-associated shape (Figure 4A). The FTT shape possessed a narrower supraspinous fossa and teres major origin site. The acromion of the FTT shape was anteroposteriorly narrower and projected slightly more laterally, and the glenoid of the FTT shape was anteverted and more superiorly inclined. The coracoid of the FTT shape was rotated anteriorly and inferiorly. The FTT shape’s scapular spine was more cranially (superiorly) oriented than the control shape’s scapular spine. The discrete metrics calculated for each shape further supported these qualitative differences observed on the thin plate spline warps (Figure 4; Table 1). Notably, glenoid version differed most substantially between shapes, with the FTT-associated shape being 6.1° anteverted relative to the control-associated shape. The FTT shape also had a larger critical shoulder angle (+3.2°), more glenoid inclination (+2.0°), a shorter (−1.1 mm) and narrower (−0.9 mm) glenoid, less acromion coverage (−2.8°), and a greater lateral acromion ratio (+3.1°) indicating relatively more acromial overhang.

**TABLE 1:** Discrete metrics calculated on the mean control shape and the mean FTT shape. The exact values are dependent on individual scapula (template) mesh used to generate warps for the mean control and mean FTT shapes, but the direction of shape changes between the control and FTT shapes would be consistent for any chosen mesh.

Feature	Control	FTT
Critical shoulder angle (°)	31.5	34.7
Glenoid inclination (°)	10.8	12.8
Glenoid version (°)	2.7	8.8
Glenoid height (mm)	31.3	30.2
Glenoid width (mm)	23.7	22.8
Acromion coverage (°)	67.4	64.6
Lateral acromion ratio (%)	31.1	34.2

### 3.2 Supraspinatus stabilizing potential for base kinematic path

**FIGURE 5:**
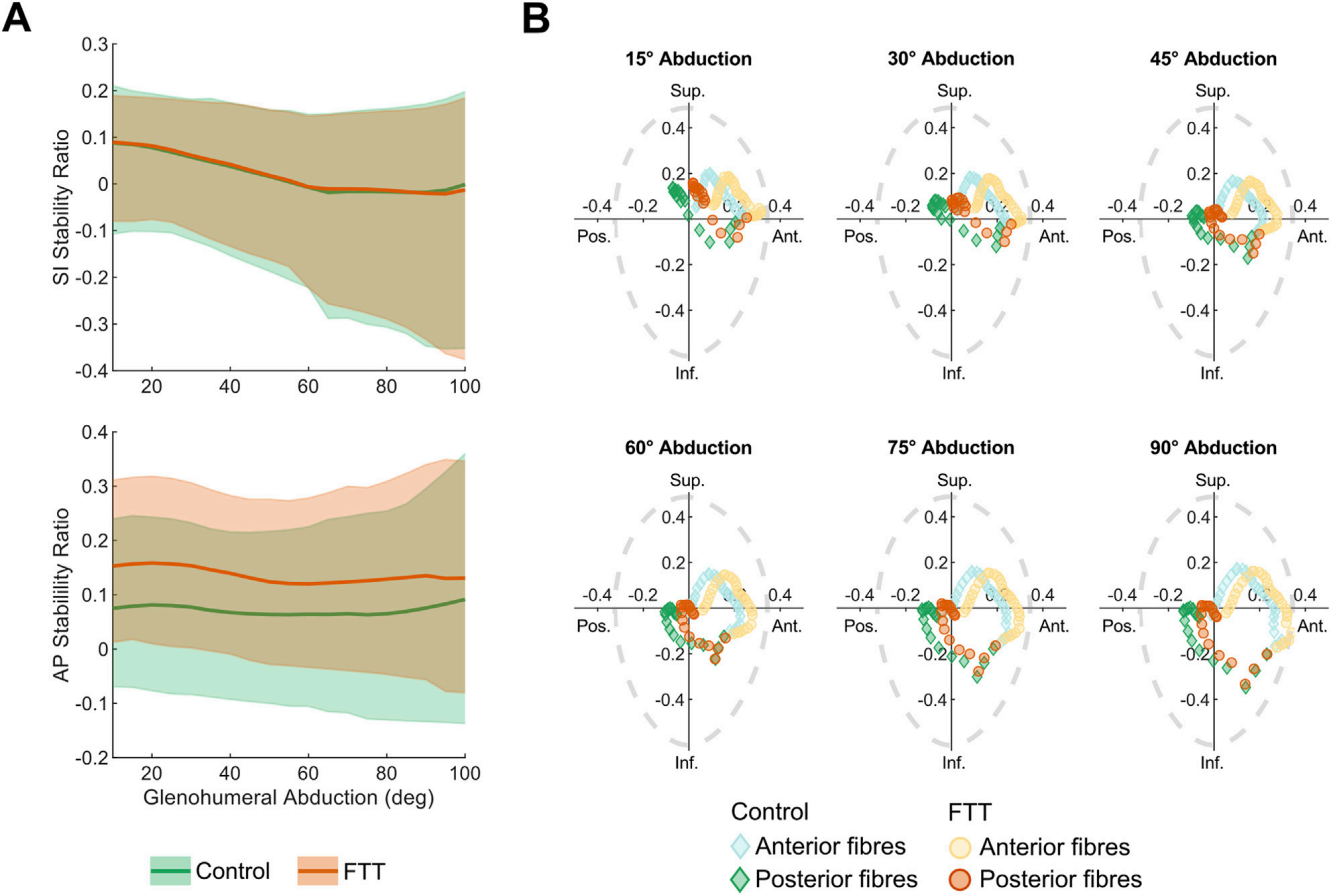
Supraspinatus stability ratios for control- and FTT-associated shapes with base abduction kinematics **(A)** SI and AP stability ratios across abduction level. The dark line is the mean and the shaded region spans the range of supraspinatus fibres **(B)** SI vs AP stability at increments spanning glenohumeral abduction. Each point represents a fibre where the light and dark points are fibres from the anterior and posterior subregions, respectively. A purely compressive line-of-action would be at the origin. For context, the dotted grey line depicts the experimentally measured glenohumeral limits of stability, where a net force acting outside of the limits of stability would cause the humerus to dislocate from the glenoid (Lippitt and Matsen, 1993). Note that these limits of stability represent a sample average, but they could vary across individuals according to glenoid and labral morphology.

For both scapula shapes, the supraspinatus’s mean SI stability ratio decreased as abduction level increased, meaning the fibres’ lines-of-action on the humerus were oriented, on average, more inferiorly as abduction increased (Figure 5A). The fibres’ stability ratios also became more variable at higher abduction levels, indicating greater superior-inferior breadth in the fibres’ lines-of-action. The mean AP stability ratios were more consistent throughout the range-of-motion, indicating an anterior line of action. In general, the anterior fibres were oriented more anteriorly and superiorly relative to the posterior fibres (Figure 5B).

At all levels of abduction, the FTT shape had higher AP stability ratios than the control shape (Figure 5). Across the range of motion, the FTT shape’s mean AP stability ratio was 0.04–0.08 higher (more anterior). This indicates that the FTT shape’s supraspinatus line-of-action was oriented more anteriorly relative to the control shape. The mean SI stability ratio was consistent across shapes and did not differ between shapes by more than 0.01 throughout the range-of-motion.

The FTT shape generally had a lower range in stability ratios across supraspinatus fibres compared to the control shape, indicating less variation in the FTT shape’s supraspinatus line-of-action. (Figure 5). The range in AP stability ratios differed most at high abduction levels, where the FTT shape’s range was 14% lower than the control shape’s range. The range in SI stability ratios differed most at low abduction levels, where the FTT shape’s range was 15% lower than the control shape’s range.

### 3.3 Supraspinatus stabilizing potential for perturbed kinematic paths

3D range-of-motion simulations estimated the kinematic neutral pose of the FTT-associated scapula to be more anterior (+2.5° in elevation plane) and more internally rotated (+2.1° in axial rotation) relative to the control-associated scapula. Therefore, we simulated perturbed kinematic paths that were more anterior (+5°, 10°), more internally rotated (+5°, 10°), or a combination of both (+5°, 10° in each) on the FTT shape.

**FIGURE 6:**
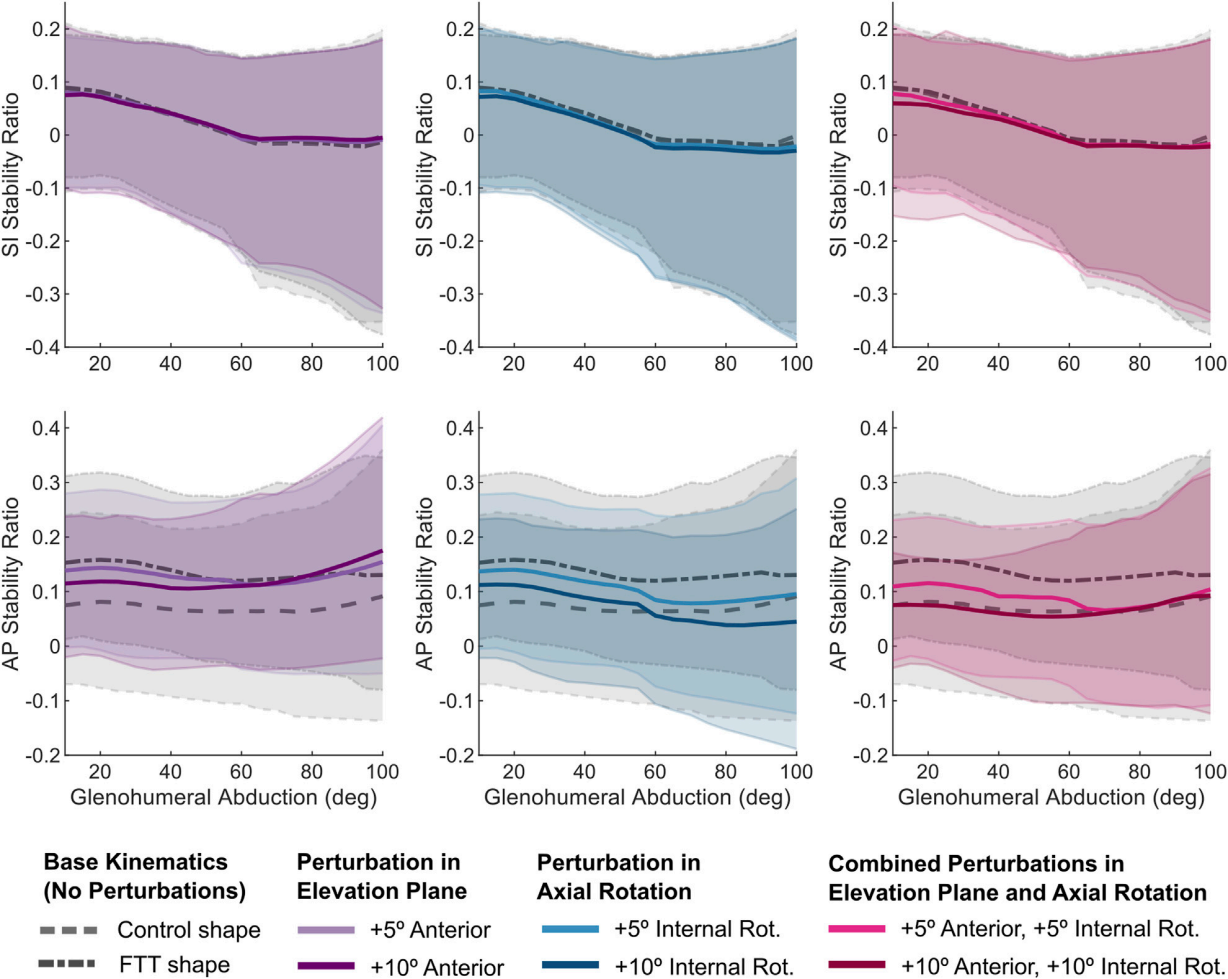
SI and AP stability ratios vs abduction level for the perturbed kinematic paths simulated on the FTT-associated shape. The dark line is the mean and the shaded region spans the range of supraspinatus fibres. For reference, the stability ratios for the base kinematic paths (for both control and FTT shapes) are plotted in grey. The combined perturbations (in pink) most effectively shifted the FTT stability ratios to resemble those of the control shape.

Independently, perturbing the elevation plane anteriorly and adding internal rotation shifted the FTT shape’s mean stability ratios inferiorly and posteriorly (Figure 6). However, the stability ratios were generally more sensitive to perturbations in internal rotation. Relative to the 5° perturbations, the 10° perturbations more effectively shifted the magnitude of the FTT stability ratios closer to the control-shape stability ratios with the base kinematics (Figure 6). However, none of the independent perturbations achieved a magnitude and pattern of the mean stability ratios throughout abduction that resembled those of the control shape.

**FIGURE 7:**
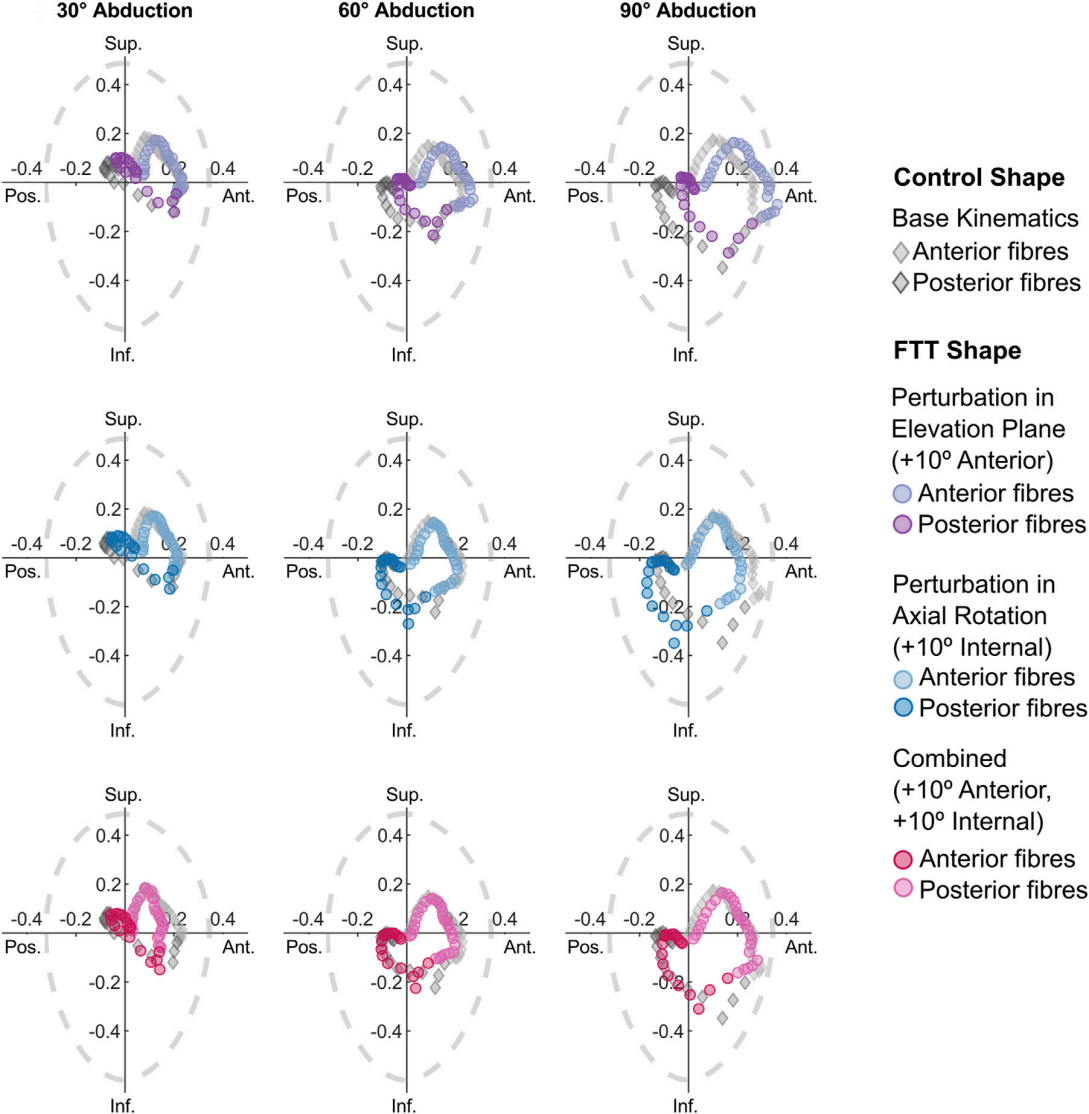
SI vs AP stability ratios for the perturbed kinematic paths simulated on the FTT shape. Each point represents one fibre. Only the stability ratios from the 10° perturbations are plotted. For reference, the stability ratios for the control shape with the base kinematic path are included as grey diamonds. The dotted grey line depicts glenohumeral limits of stability (Lippitt and Matsen, 1993).

The 10° combined perturbation (+10° anterior elevation plane, +10° internal rotation) best achieved FTT shape stability ratios that replicated the control shape’s stability ratios (Figure 6). Specifically, the mean AP stability ratio of the FTT shape with the 10° combined perturbation closely resembled the mean AP stability ratio of the control shape across the entire range of glenohumeral abduction (Figure 6). However, the range in AP stability ratios remained substantially narrower in the FTT shape compared to the control shape - particularly at low abduction levels where there was a 36% reduction in range (Figures 6, 7). Therefore, although the combined perturbation achieved the desired shift in the mean AP stability ratios, the breadth of the fibres’ lines-of-action was still narrower in the FTT shape compared to the control shape (Figure 7; bottom row). The combined perturbation also shifted the SI stability ratios inferiorly relative to the control shape, but this shift was less than 0.03 throughout the range of motion (Figure 6).

## 4 Discussion

In this study, we combined a 3D scapula shape model, kinematic simulations of the glenohumeral joint, and a morphable finite-element model to investigate how tear-associated scapula morphology alters the stabilizing potential of the supraspinatus. Relative to asymptomatic controls, individuals with symptomatic FTTs possessed a suite of geometric differences including narrower supraspinous fossae and anteverted glenoids. We found that, for the same planar abduction path, FTT-associated scapula shapes shifted the supraspinatus stability ratios anteriorly compared to the control-associated shape. When the FTT shape’s abduction path was perturbed anterior in elevation plane and internally rotated, its stability ratios closely resembled those of the control-associated shape; however, the FTT shape’s narrower breadth of the supraspinatus line-of-action persisted. The magnitude of perturbation required to modulate stability ratios exceeded the magnitude of shape changes between the control-associated and FTT-associated shapes, indicating the 3D shape differences altered the supraspinatus’ function in a complex manner.

Our 3D PLSDA model confirmed previous FTT-associated features and revealed new injury-relevant features. Consistent with previous studies, the FTT shape possessed a higher critical shoulder angle (Cherchi et al., 2016; Moor et al., 2013) and a more superiorly inclined glenoid (Hughes et al., 2003; Lee et al., 2020) – two features that increase the destabilizing action of the deltoid and increase the load required of the rotator cuff during abduction (Gerber et al., 2014; Moor et al., 2016; Oswald et al., 2024; Viehöfer et al., 2015). This mechanism was further supported by the FTT shape’s higher lateral acromion ratio, which indicated a more laterally projected acromion. However, our 3D landmark-based approach also revealed changes to the glenoid and muscle attachment sites that could influence the lines-of-action and stabilizing potential of the rotator cuff muscles themselves. For instance, the supraspinous fossa and teres major attachment sites were narrower in the FTT shape. Our model showed that the narrower supraspinous fossa reduced the breadth of the supraspinatus muscle; thus, a similar effect may occur at the teres major. The FTT shape also had a shorter and narrower glenoid, which could reduce the glenohumeral limits of stability - the range within which the net force on the humerus is considered stable (Lippitt and Matsen, 1993).

The most striking shape difference between the FTT and control groups was in glenoid version, as the FTT shape was substantially more anteverted (Table 1). This finding differs from previous work indicating no difference (Kandemir et al., 2006) or more retroversion in rotator cuff tear patients (İncesoy et al., 2021; Maalouly et al., 2020; Tokgoz et al., 2007). This discrepancy may be explained by the difference in version calculated from 2D axial slices vs the 3D approach used here (Budge et al., 2011). Our finding may also reflect a difference in the tear initiation site across the FTT patients in each study. Tétrault et al. found that glenoid anteversion is correlated with the posterior cuff tears involving the infraspinatus and/or supraspinatus, while glenoid retroversion is correlated with anterior cuff tears involving the subscapularis and/or supraspinatus (Tétreault et al., 2004). While the anatomical origin of the FTTs of patients in this study is unknown, it is possible that the mean glenoid anteversion found here reflects a tendency towards posterior cuff tear initiation in our sample.

A major finding of our study is that for the same abduction path, the FTT-shape’s supraspinatus fibres acted more anteriorly and less compressively compared to the control shape (indicated by higher AP stability ratios). While the magnitude of the mean supraspinatus stability ratios was generally low (<0.15) for both shapes - confirming the supraspinatus’ tendency to compress the humeral head into the glenoid–this anterior shift may have implications for the muscle’s stabilizing potential in FTT-associated shapes. It is important to consider the stabilizing role of the supraspinatus when interpreting these implications. During scapular plane abduction, the deltoid imparts a destabilizing superior shear force on the humerus that decreases as the arm is elevated (Ackland and Pandy, 2009; Mulla et al., 2019). The concavity-compression mechanism posits that rotator cuff muscles co-contract to resist the large shear forces cause by prime movers like the deltoid (Lippitt et al., 1993). In this case, the supraspinatus’s ideal SI and AP stability ratios would be zero, indicating a purely compressive line-of-action. However, Sangwan et al. reported limited evidence of co-contraction across the rotator cuff. They proposed an alternate mechanism where the muscles act in isolation or as sub-units to resist shear translations in a direction-specific manner (Sangwan et al., 2015). If the supraspinatus were to directly resist the deltoid’s superior shear force, it would ideally act inferiorly (negative SI ratio) and be balanced anteriorly-posteriorly (AP stability ratio close to zero). Whether the stabilizing mechanism is compressive, direction-specific, or both, the FTT shape’s stability ratios are unbalanced antero-posteriorly and likely problematic for either mechanism. The FTT shape’s line-of-action is almost entirely anterior across its fibres, with only the most posterior fibres having the potential to provide a posterior stabilizing force (Figure 5). This may place a higher demand on the posterior fibres for providing a balanced stabilizing force and/or limit the supraspinatus’ potential to stabilize the humerus against anterior shear forces. This interpretation is consistent with the finding that individuals with anteverted glenoids have a higher prevalence of tears involving the supraspinatus and infraspinatus tendons, and supports the hypothesis that an anteverted glenoid places a higher strain on fibres of the posterior rotator cuff. (Tétreault et al., 2004).

Kinematically perturbing the abduction path partially modulated the shape-related changes in supraspinatus stabilizing potential. Perturbations that accommodated the differences in the shapes’ kinematic neutral poses shifted the FTT shape’s stability ratios posteriorly such that the mean stability ratios overlapped those of the control shape (Figure 6). This finding indicates that individuals may adapt their glenohumeral kinematics according to their unique anatomy to modulate shape-related differences in muscle function. Given that an individual’s environment and daily tasks likely demand specific arm postures and motions, it may not be practical or reasonable to expect individuals to adapt their overall humerothoracic motion. However, the human shoulder girdle is highly mobile and kinematic degrees of freedom across multiple articulations provide us with the flexibility of a variety of joint configurations to execute a single arm posture (Larson, 2009; Ludewig et al., 2009). Therefore, individuals could more likely achieve their shape-specific glenohumeral patterns and execute the same humerothoracic motion by adjusting their scapular posture and kinematics. For instance, individuals can achieve a more anterior and internally rotated glenohumeral pose by adding scapular external rotation at low abduction levels, and a combination of scapular external rotation and posterior tilt at high abduction levels. This has implications for the diagnosis and treatment of movement impairments. Insufficient posterior tilt and excessive internal rotation of the scapula are considered pathological movement patterns that contribute to shoulder pain (Ludewig et al., 2022). Our findings suggest that promoting scapular posterior tilt and external rotation through physiotherapy intervention can facilitate the supraspinatus’ stabilizing function. However, our results also suggest that optimal movement patterns vary with bony anatomy. Thus, strictly classifying movement outside a narrow range of expected movement as “abnormal” or “pathological” may not capture the variation in healthy motion across individuals (Caldwell et al., 2007; Ludewig et al., 2022).

Interestingly, the magnitude of perturbations required to modulate shape-related differences (10°) greatly exceeded the magnitude of difference in kinematic neutral poses (2.1°–2.5°) and the difference in glenoid version (6.1°). This indicates that the kinematic perturbations are not simply correcting for an offset introduced by factors such as coordinate system definition, but rather complex changes in muscle geometry that occur due to the 3D shape differences. This speculation is further supported by the fact that, even when the mean stability ratios were overlapping, the range in the FTT shape’s stability ratios was much lower than in the control shape (Figures 6, 7). Therefore, while perturbations could potentially correct for changes to muscle function resulting from glenoid anteversion in the FTT shape, they could not overcome the effect of the narrower supraspinous fossa on reducing the breadth of the supraspinatus’s line-of-action. This narrower breadth may inhibit the supraspinatus’s ability to modulate its resultant line-of-action in response to varying destabilizing forces.

This study complements previous work that found tear-associated scapula morphology alters the mechanical advantage of the supraspinatus (Lee et al., 2020). While we focused here on the supraspinatus’s role in stabilizing the humeral head, it also contributes to shoulder abduction moment (Reed et al., 2013). Together, these studies found that the supraspinatus’s abduction moment arm and stabilizing potential are inhibited in FTT-associated scapula shapes at low levels of abduction (Lee et al., 2020). Since most activities of daily living involve abduction at low levels and the deltoid’s destabilizing shear force is highest in this range (Mulla et al., 2020), these findings highlight multiple ways in which FTT-associated morphology may elevate strain in the supraspinatus tendon required for arm-raising.

Our morphable, finite-element approach allowed us to estimate stability ratios across the breadth of the supraspinatus. The magnitude of the mean supraspinatus stability ratios was consistent with previously reported cadaveric (Ackland and Pandy, 2009; Lee et al., 2000) and computational studies (Lee et al., 2000; Mulla et al., 2020). While previous approaches divided stability ratios by the centres of distinct subregions (Ackland and Pandy, 2009) or perturbed attachment sites with probabilistic modelling (Mulla et al., 2019), our approach reported stability ratios across the breadth of the sheet-modelled supraspinatus spanning the entire 3D attachment site, similar to (Webb et al., 2014). We found the posterior fibres exhibited more compressive lines-of-action (lower stability ratios) while the anterior fibres exhibited more anterior lines-of-action (higher AP stability ratios), and this breadth in stability ratios differed substantially between control- and FTT-associated shapes.

Our study had several limitations. First, our model was limited to the supraspinatus, but all rotator cuff muscles contribute to stability and the FTT shape involved differences several rotator cuff attachment sites. While we predict the glenoid anteversion would cause an anterior shift in all muscle lines-of-action, further modelling will quantitively examine whether this is the case. Second, our sample is unbalanced, with relatively fewer individuals with FTTs than controls. While we assessed our PLSDA model through internal cross-validation, external validation on new individuals could assess whether our model is generalizable outside of the present sample. Third, we chose to simulate planar abduction, but alterations to the supraspinatus’s stabilizing potential in other motions, such as flexion or horizontal ab-adduction, could also contribute to tendon overload. Fourth, our Artisynth model forced the supraspinatus to remain in tension throughout the entire range-of-motion. This may not be the case–particularly at high abduction levels where supraspinatus activity decreases (Escamilla et al., 2009). Finally, we mapped our model to theoretical scapula shapes representing the mean shapes of the FTT and control groups. While this approach allowed us to deterministically test the effect of injury-relevant shape modes, person-specific models (Oswald et al., 2024) may offer additional insight into how supraspinatus stabilizing potential is affected by unique combinations of shape features that are not captured along a single shape axis. Our landmark-based shape model also prevented us from capturing morphological details such as injury-related differences in curvature in the glenoid fossa or supraspinous fossa. Shape models based on correspondence across dense landmarks or mesh vertices could overcome this limitation (Cates et al., 2017; Sharif-Ahmadian et al., 2023). However, these automated approaches are challenged by the scapula’s thin blade and convex curvature, and further validation is required to implement them.

In conclusion, this study presents an approach for mapping 3D shape-related changes to rotator cuff function and exploring the influence of kinematic adjustments. We found that an anteverted glenoid, among other shape changes, shifts the supraspinatus line-of-action anteriorly–potentially inducing elevated strain in the posterior fibres–and that this shift could be partially modulated with kinematic perturbations that exceeded the magnitude of observed shape differences. Importantly, we present a framework that enables the analysis of supraspinatus lines-of-action at any 3D rotational pose across scapula shapes. Our approach could be expanded to include additional rotator cuff muscles and investigate how stabilizing potential is altered in other injury-relevant motions.

## Data Availability

The data analyzed in this study is subject to the following licenses/restrictions: One subset of the scapula meshes were acquired from an open-access dataset (Kolz et al., 2020; 10.5281/zenodo.4289455). One subset of the scapula meshes were provided by Dr. Michael Bey (Henry Ford Health). We do not have permission to share these data. One subset of the scapula meshes were collected by Rebekah Lawrence (co-author). These data were collected as part of an ongoing NIH grant, and will be publically available at the end of the grant. The data and MATLAB code required to plot the results are available as Supplementary Material. The thin plate spline warps for the control- and tear-associated scapulae are provided as STLs.

## Supplementary material

Supplementary Data Sheet S1 Supplementary tables, figures, and a description of the data and code provided in Data Sheet 2.

Supplementary Data Sheet S2 The data and MATLAB code required to the plot the results.
