# Supplementary material for: The stabilizing potential of the supraspinatus is inhibited in tear-associated scapula shapes but can be modulated by kinematic adjustments
Source: Front Bioeng Biotechnol. 2025 Mar 31;13:1505015. doi: 10.3389/fbioe.2025.1505015 (PMC11994605; doi:10.3389/fbioe.2025.1505015)
Supplement: Supplementary file 1 [file DataSheet1.pdf]

This document contains supplementary tables, figures, and a description of the data to support the publication:

Lee ECS, Young NM, Li EY, Lawrence RL and Rainbow MJ (2025). The stabilizing potential of the supraspinatus is inhibited in tear-associated scapula shapes but can be modulated by kinematic adjustments. *Front. Bioeng. Biotechnol*.13:1505015. doi:10.3389/fbioe.2025.1505015

Supplementary Tables and Figures

Table S 1: Descriptions of discrete metrics.

Feature	Description
Critical shoulder angle (°)	The angle between (a) superior-inferior glenoid vector and (b) a vector between the inferior glenoid and lateral acromion, in the sagittal plane.
Glenoid inclination (°)	The angle between the scapula's superior-inferior axis and the glenoid's superior axis, in the frontal plane. Superior inclination is positive.
Glenoid version (°)	The angle between (a) a vector perpendicular to the glenoid's anterior axis, in the transverse plane and (b) the scapula's lateral axis. Anteversion is positive and retroversion is negative.
Glenoid height (mm)	The height of the glenoid along its superior-inferior axis.
Glenoid width (mm)	The width of the glenoid along its anterior-posterior axis.
Acromion coverage (°)	The angle between (a) a vector oriented from the glenoid centre to the anterior aspect of the acromion and (b) a vector oriented from the glenoid centre to the posterior aspect of the acromion, in the sagittal plane.
Lateral acromion ratio (%)	The ratio of the lateral acromion extension (the distance between the base of the coracoid and the lateral acromion) to the scapular body width (the distance between the root of the scapular spine and the base of the coracoid).

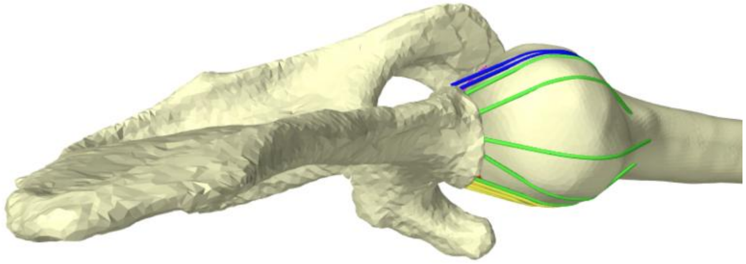

Figure S 1: An example of wrapping error that can occur when fibres are modelled independently. This example is for the inferior glenohumeral ligament, where fibres spread apart around the convex surface of the humeral head. The finite element sheets provide axial tension between fibres that prevents fibres from spread apart.

Description of Data

DataAndCode.zip contains the data and script required to reproduce the results figures from the manuscript (Figures 3, 5-7). It also contains the 3D scapula meshes as STL files.

- **Scapula_control.stl** and **scapula_tear.stl** are thin plate spline warps of the control-associated and tear-associated scapula shapes, respectively. They correspond to the shape coordinates associated with a y score of y = 0 (for control) and y = 1 (for tear). These are the meshes that were used in the Artisynth model for the control- and tear-associated supraspinatus lines-of-action.

- **recreatePlots.m** is a MATLAB script that will reproduce the plots. For required data and subfunctions to be in the MATLAB path, run this script from the folder where the contents from *DataAndCode.zip* were extracted.

- **PLSDA_Data.mat** is a MATLAB structure containing variables associated with the PLSDA model.
  - **nsubs:** sample size.
  - **labels:** Labels for whether the individual was asymptomatic (Asym) or symptomatic (Sym)
  - **PctExplained:** The percent of shape variation (row 1) and label variation (row 2) explained by each latent variable (LV) or shape mode.
  - **Xscores:** The latent variable (shape mode) scores for each individual.
  - **B_accuracy, F1 score,** and **Gmean:** Performance metrics for final PLSDA model.
  - **[Metric]_LVs:** Performance metrics for models with increasing number of retained latent variables (shape modes) retained (from 1 to 14).
  - **[Metric]_rand:** Performance metrics for models generated from random labels.
  - **nLV:** Optimal number of retained latent variables.
  - **YL:** The loading vector of the shape modes (defining the discriminant axis).
  - **GrpTF:** Whether the individual has a symptomatic tear (1) or is an asymptomatic control (0).
  - **control_shape_coords:** The 3D shape coordinates reconstructed the control-associated shape (used to generate *scapula_control.stl*).
  - **tear_shape_coords:** The 3D shape coordinates reconstructed the tear-associated shape. (used to generate *scapula_tear.stl*).
  - **shapeCoords:** The 3D shape coordinates for each individual in the sample (following Procrustes analysis for scaling and alignment).

- **Fibre_Data.mat** is a MATLAB structure containing variables associated with the supraspinatus fibre lines-of-action computed from the Artisynth model. \\
  - The first field layer corresponds to the shape (**pls00** for the control shape and **pls100** for the tear shape).

- The second field layer corresponds to the kinematic path, following the naming convention **PerturbedAbduction_plaX_rotnYY**, where X is the plane of elevation (in degrees), and YY is the amount of internal rotation (in degrees). Each kinematic field then contains subfields for **SUP** (the superficial supraspinatus sheet), **SUP2** (the deep supraspinatus sheet), and **SUPall** (both sheets combined into one variable). Each sheet variable then has the following subfields describing the supraspinatus fibres for the given shape and motion path:
    - **LOA_glen** (50x3x20). The 3D line of action of each supraspinatus fibre (for rows 1 to 50) for each frame in the simulation (20 frames). The line of action is resolved to the glenoid-based coordinate system.
    - **stabilityRatio_SI** (50x20). The superior-inferior stability ratio of each supraspinatus fibre (for rows 1 to 50) for each frame in the simulation.
    - **stabilityRatio_AP** (50x20). The anterior-posterior stability ratio of each supraspinatus fibre (for rows 1 to 50) for each frame in the simulation.
    - **abduction** (20x1). The abduction level, in degrees, at each frame of the simulation.
