# Supplementary material for: The stabilizing potential of the supraspinatus is inhibited in tear-associated scapula shapes but can be modulated by kinematic adjustments
Source: Front Bioeng Biotechnol. 2025 Mar 31;13:1505015. doi: 10.3389/fbioe.2025.1505015 (PMC11994605; doi:10.3389/fbioe.2025.1505015)
Supplement: Supplementary file 2 [file DataSheet2.zip › Subfunctions/arrow3D_pub/html/demoArrow3D.html]

arrow3D - creates a single volumized arrow based on a cylinder/cone combination


 


# arrow3D - creates a single volumized arrow based on a cylinder/cone combination

## Contents

- Basic Setup
- Enhancing Visual Appearance
- Stem Ratio Options
- arrow3D handle Options
- Credits

## Basic Setup

```
    arrow3D([0,0,0] ,[1,2,3]);
    hold on;   axis equal;   xlabel('X'); ylabel('Y'); zlabel('Z');
    title('Basic arrow3D call');
```

## Enhancing Visual Appearance

```
    lighting phong;
    camlight right;
    title('');
```

## Stem Ratio Options

```
    subplot(131);
    arrow3D([0,0,0], [0,0,3] , 'r', 0.25);
    hold on;   axis equal;   xlabel('X'); ylabel('Y'); zlabel('Z');
    lighting phong;
    camlight right;
    title('Stem Ratio = 0.25');

    subplot(132);
    arrow3D([0,0,0], [0,0,3], 'g', 0.5);
    hold on;   axis equal;   xlabel('X'); ylabel('Y'); zlabel('Z');
    lighting phong;
    camlight right;
    title('Stem Ratio = 0.5');

    subplot(133);
    arrow3D([0,0,0], [0,0,3], 'b', 0.75);
    hold on;   axis equal;   xlabel('X'); ylabel('Y'); zlabel('Z');
    lighting phong;
    camlight right;
    title('Stem Ratio = 0.75');
```

## arrow3D handle Options

The form of the arrowHandle is the same as 'surf'. arrowHandle = [arrowStem, arrowHead]

```
    subplot(131);
    hold off;
    arrow3D([0,0,0], [0,0,3], 'r');
    hold on;   axis equal;   xlabel('X'); ylabel('Y'); zlabel('Z');
    lighting phong;
    camlight right;

    subplot(132);
    hold off;
    arrowHandle = arrow3D([0,0,0], [0,0,3], 'r');
    hold on;   axis equal;   xlabel('X'); ylabel('Y'); zlabel('Z');
    set(arrowHandle(1), 'FaceColor', 'c');
    lighting phong;
    camlight right;

    subplot(133);
    hold off;
    arrowHandle = arrow3D([0,0,0], [0,0,3], 'r');
    hold on;   axis equal;   xlabel('X'); ylabel('Y'); zlabel('Z');
    set(arrowHandle(1), 'FaceColor', 'c');
    set(arrowHandle(2), 'FaceColor', 'b');
    lighting phong;
    camlight right;
```

## Credits

Author: Shawn Arseneau

Created: September 14, 2006

Published with MATLAB® 7.0.1
