# Supplementary figures and images for: The stabilizing potential of the supraspinatus is inhibited in tear-associated scapula shapes but can be modulated by kinematic adjustments
Source: Front Bioeng Biotechnol. 2025 Mar 31;13:1505015. doi: 10.3389/fbioe.2025.1505015 (PMC11994605; doi:10.3389/fbioe.2025.1505015)

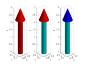

Supplement: Supplementary file 2 [file DataSheet2.zip › Subfunctions/arrow3D_pub/html/demoArrow3D.png]

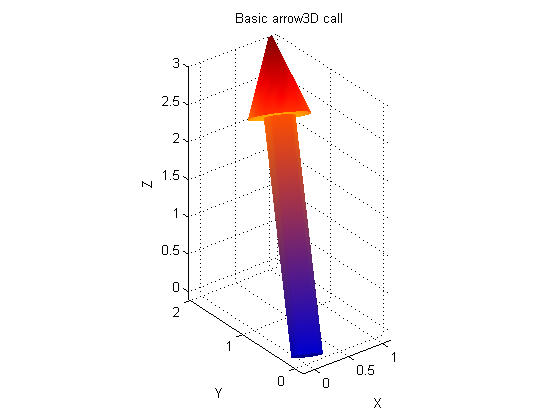

Supplement: Supplementary file 2 [file DataSheet2.zip › Subfunctions/arrow3D_pub/html/demoArrow3D_01.png]

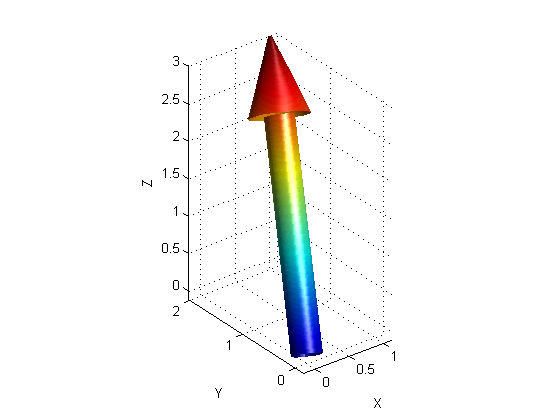

Supplement: Supplementary file 2 [file DataSheet2.zip › Subfunctions/arrow3D_pub/html/demoArrow3D_02.png]

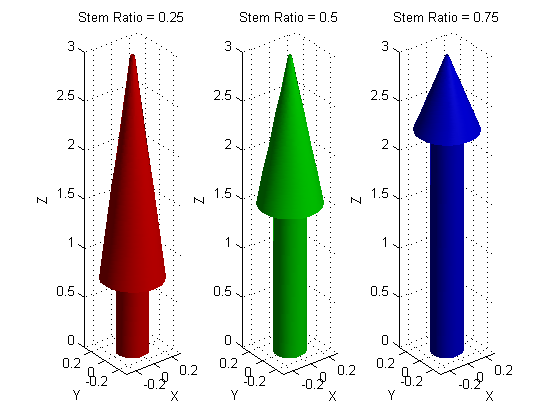

Supplement: Supplementary file 2 [file DataSheet2.zip › Subfunctions/arrow3D_pub/html/demoArrow3D_03.png]

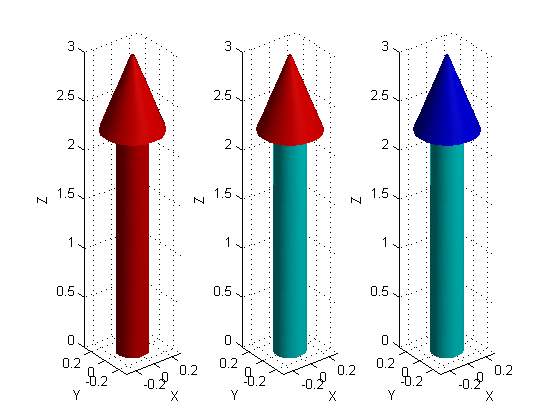

Supplement: Supplementary file 2 [file DataSheet2.zip › Subfunctions/arrow3D_pub/html/demoArrow3D_04.png]

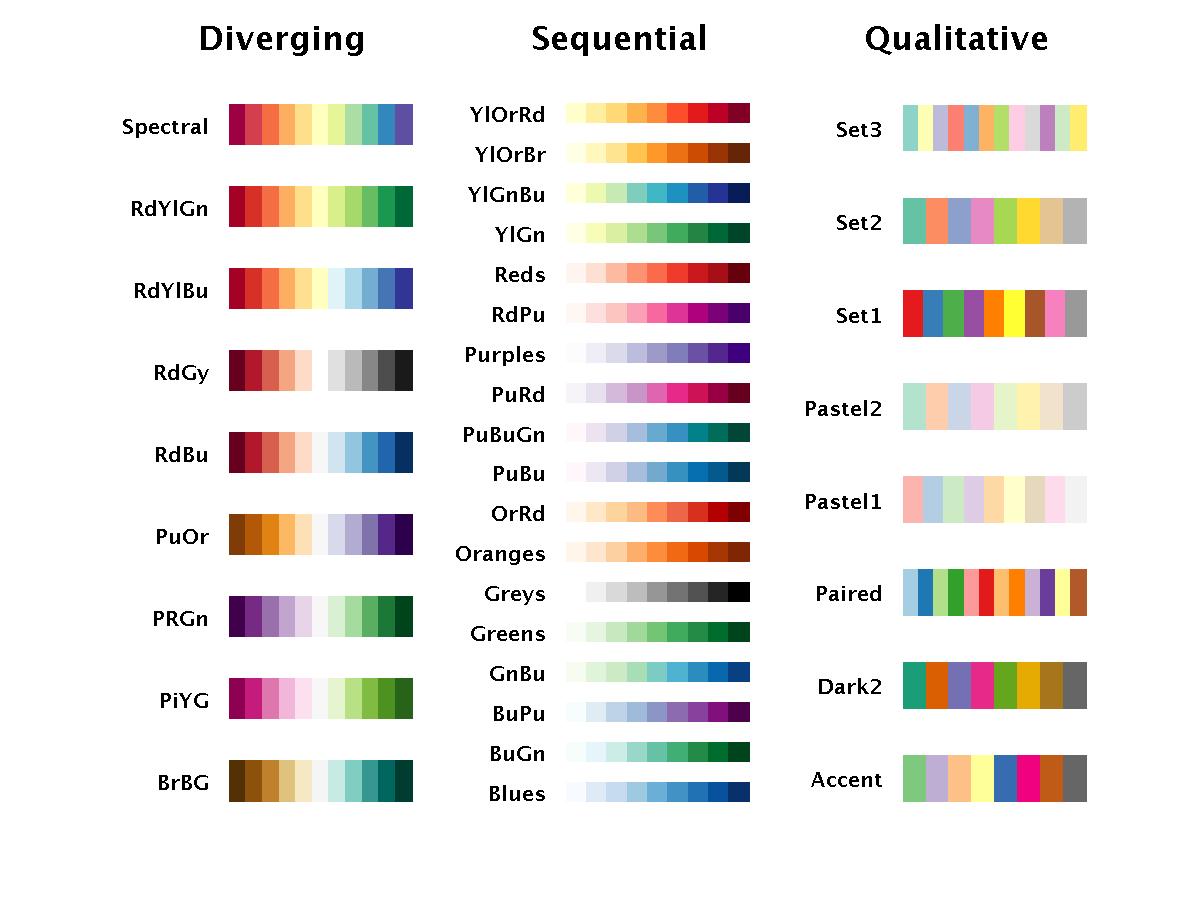

Supplement: Supplementary file 2 [file DataSheet2.zip › Subfunctions/cbrewer/cbrewer_preview.jpg]
